# Application of deep learning reconstruction of ultra-low-dose abdominal CT in the diagnosis of renal calculi

**DOI:** 10.1186/s13244-022-01300-w

**Published:** 2022-10-08

**Authors:** Xiaoxiao Zhang, Gumuyang Zhang, Lili Xu, Xin Bai, Jiahui Zhang, Min Xu, Jing Yan, Daming Zhang, Zhengyu Jin, Hao Sun

**Affiliations:** 1 Department of Radiology, State Key Laboratory of Complex Severe and Rare Disease, Peking Union Medical College Hospital, Peking Union Medical College, Chinese Academy of Medical Sciences, Shuaifuyuan No.1, Wangfujing Street, Dongcheng District, Beijing, 100730 China; 2 National Center for Quality Control of Radiology, Beijing, China; 3 Canon Medical System (China), No.10, Jiuxianqiao North Road, Chaoyang District, Beijing, 100024 China

**Keywords:** Abdominal CT, Ultra-low-dose CT, Renal calculi, Deep learning reconstruction

## Abstract

**Background:**

Renal calculi are a common and recurrent urological disease and are usually detected by CT. In this study, we evaluated the diagnostic capability, image quality, and radiation dose of abdominal ultra-low-dose CT (ULDCT) with deep learning reconstruction (DLR) for detecting renal calculi.

**Methods:**

Sixty patients with suspected renal calculi were prospectively enrolled. Low-dose CT (LDCT) images were reconstructed with hybrid iterative reconstruction (LD-HIR) and was regarded as the standard for stone and lesion detection. ULDCT images were reconstructed with HIR (ULD-HIR) and DLR (ULD-DLR). We then compared stone detection rate, abdominal lesion detection rate, image quality and radiation dose between LDCT and ULDCT.

**Results:**

A total of 130 calculi were observed on LD-HIR images. Stone detection rates of ULD-HIR and ULD-DLR images were 93.1% (121/130) and 95.4% (124/130). A total of 129 lesions were detected on the LD-HIR images. The lesion detection rate on ULD-DLR images was 92.2%, with 10 cysts < 5 mm in diameter missed. The CT values of organs on ULD-DLR were similar to those on LD-HIR and lower than those on ULD-HIR. Signal-to-noise ratio was highest and noise lowest on ULD-DLR. The subjective image quality of ULD-DLR was similar to that of LD-HIR and better than that of ULD-HIR. The effective radiation dose of ULDCT (0.64 ± 0.17 mSv) was 77% lower than that of LDCT (2.75 ± 0.50 mSv).

**Conclusion:**

ULDCT combined with DLR could significantly reduce radiation dose while maintaining suitable image quality and stone detection rate in the diagnosis of renal calculi.

## Key points

- Ultra-low-dose CT could be used to detect kidney stones.
- Ultra-low-dose CT could be used to detect abdominal lesion.
- DLR could maintain image quality when the radiation dose was reduced to sub-millisieverts.

## Background

Renal calculi are a common and often recurrent urinary disease with an incidence rate of 10–15%, which is increasing year by year [1, 2]. Most renal calculi are asymptomatic, but 10–25% of patients suffering from renal calculi require intervention due to renal colic or hematuria [3]. When an asymptomatic renal calculus grows in size, which can lead to urinary-tract blockage or recurrent infections, lithotripsy or nephrolithotomy is required [4]. Abdominal computed tomography (CT) imaging is the most accurate method for confirming and monitoring suspected renal calculi. The sensitivity and specificity of CT for renal calculi are 95% and 98%, respectively, and CT performs better than kidney–ureter–bladder (KUB) radiography and ultrasound [5–7]. In addition, CT can more accurately evaluate the positions and sizes of renal calculi and facilitate subsequent treatments [5–7]. The potential risk of ionizing radiation caused by multiple CT scans limits the use of CT to some extent [8, 9]. However, decreasing the radiation dose increases the amount of image noise. Therefore, lowering the radiation dose while maintaining high CT image quality for diagnosis has long been a clinical goal [10, 11].

Many studies show that a combination of low-kilovoltage scanning and iterative reconstruction can be applied to achieve low-dose CT (LDCT) imaging for renal-calculus detection, and that diagnostic efficiency is comparable between LDCT and conventional dose CT [12–14]. To date, LDCT has been routinely used for the detection and confirmation of renal calculi [14]. However, the ability of iterative reconstruction to reduce noise decreases when the radiation dose is lowered to sub-millisievert ultra-low doses [15–19]. The latest deep learning reconstruction (DLR) algorithm for images, intend to optimize image quality, has been commercialized for the first time to accompany the Canon CT System (Advanced Intelligent Clear-IQ Engine; Canon Medical, Otawara, Japan) [20]. DLR integrates a deep convolutional neural network into the reconstruction process. High-quality model-based iterative reconstruction images data is performed on deep learning methods to learn signal, noise, and artifact characteristics for differentiation, and this methodology results in good recognition and decreased noise [21, 22]. Low-noise images can be obtained with DLR, which greatly improves image quality in low- to ultra-low-dose CT (ULDCT) scans and does not increase post-reconstruction processing time [20, 23–26]. In this study, we evaluated whether the DLR algorithm could maintain image quality and diagnostic capability in abdominal-ULDCT-based diagnosis of renal calculi.

## Methods

### Study population

This prospective study was approved by the Medical Ethics Committee of our institution (No. HS-2427). We obtained written informed consent for both abdominal LDCT and ULDCT from each enrolled patient. Patients with suspected renal calculi were recruited from November to December 2020. All patients underwent Canon CT scans. Exclusion criteria were age < 18 years and a history of abdominal or pelvic implantation, such as an arterial stent or artificial hip joint. No patients were excluded.

### Image acquisition and reconstruction

The Aquilion ONE Genesis CT system (Canon Medical) was used to acquire images. The Rotation speed was 0.5 s/round, pitch was 0.813, and scan area ranged from the apex of the liver to the bifurcation of the bilateral common iliac arteries. The scan voltage used for both low and ultra-low doses was 100 kV. Tube current were adjusted automatically. The low-dose noise index was the standard setting (7.5), and the ultra-low-dose noise index was the low-dose setting (20). LDCT images were reconstructed with hybrid iterative reconstruction (HIR, Adaptive Iterative Dose Reduction 3-Dimensional, [AIDR3D]) (LD-HIR). ULDCT images were reconstructed with HIR (ULD-HIR) and DLR (ULD-DLR). Five-millimeter images were uploaded to the picture-archiving and communication system (PACS) for unified analysis.

### Detection of calculi and abdominal lesions

A radiologist with 3 years’ working experience recorded the numbers and positions of renal calculi in images of all 3 groups and measured the diameter of each renal calculus twice to calculate a mean value. The radiologist was blinded to the groupings of images, and the images were presented in random order. LD-HIR image was used as the reference to calculate the renal-calculus detection rate from the ULD-HIR and ULD-DLR images. The same radiologist evaluated types and numbers of lesions in solid abdominal organs and measured their diameters twice to calculate the means.

### Objective evaluation of image quality

**Fig. 1:**
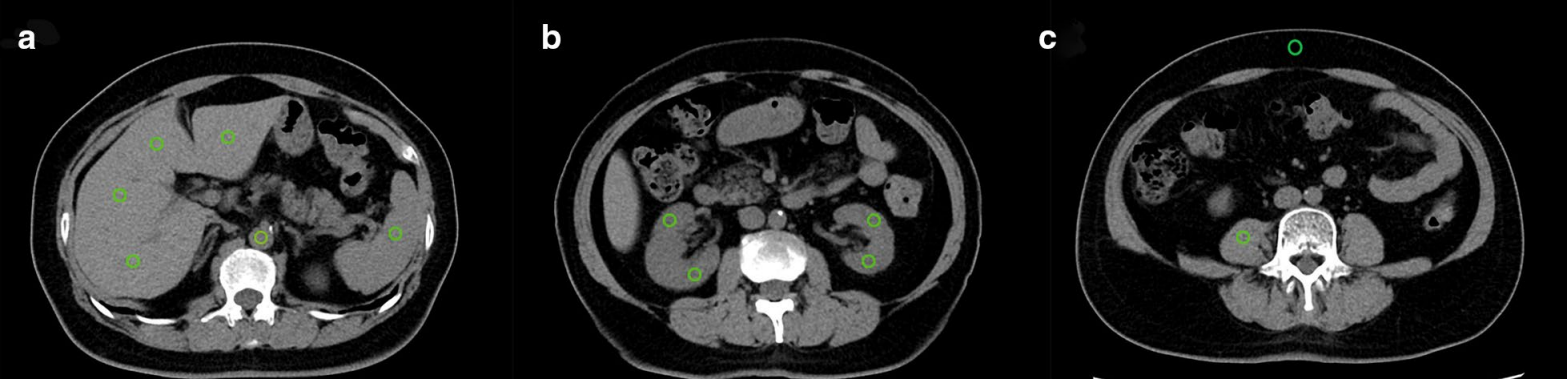
ROIs were placed on the liver, spleen, aorta, kidneys, right psoas muscle, and abdominal-wall subcutaneous fat to measure CT values and noise of various abdominal organs and to evaluate the objective image quality. **a** shows ROIs located in the liver, spleen, and aorta. **b** shows ROIs located in the kidneys. **c** shows ROIs located in the right psoas muscle and anterior abdominal-wall subcutaneous fat

Radiologist A performed quantitative analysis of cross-sectional images (section thickness, 5 mm). To measure CT value, noise (SD; standard deviation of the CT value) and signal-to-noise ratio (SNR; mean attenuation/SD), we delineated regions of interest (ROIs) in the liver, spleen, aorta, both kidneys, anterior abdominal-wall subcutaneous fat, and right psoas muscle on LD-HIR, ULD-HIR, and ULD-DLR images (Fig. 1). ROIs were kept consistent across all three groups. Those ROIs on the liver, spleen, and aorta were at the level of the hepatic hilum; those on both kidneys were at the level of the renal hilum; and the ROIs of the right psoas muscle and anterior abdominal-wall subcutaneous fat were at the level of the fourth lumbar vertebra. We placed 4 and 2 ROIs in the liver and kidneys, respectively, and calculated the means. One ROI was placed in the spleen, aorta, right psoas muscle, and subcutaneous fat each, and triplicate measurements were taken to calculate the means. ROI size for both kidneys was maintained at 0.4–0.5 cm^2^, while ROI size for all other organs was maintained at 0.8–1.0 cm^2^. Contrast-to-noise ratios (CNRs) for the liver, spleen, kidneys, and aorta were calculated by the following formula:

$${\text{CNR}}_{{{\text{organ}}}} = \left( {{\text{CT}}_{{{\text{organ}}}} - {\text{CT}}_{{\text{psoas muscle}}} } \right)/{\text{total image noise}}$$

where CT_organ_ is the CT value of the organ of interest, CT_psoas muscle_ is the mean CT value of the right psoas muscle, and total image noise is the SD of subcutaneous fat in the anterior abdominal wall [27].

### Subjective evaluation of image quality

Radiologists A and B, who had, respectively, 3 and 8 years of working experience and were blinded to the image groupings, performed 5-point Likert scoring of LD-HIR, ULD-HIR, and ULD-DLR images that were presented to them in random order. Scoring criteria were as follows: (1) extremely poor image quality, rendering diagnosis impossible; (2) poor image quality with serious noise; (3) medium image quality, sufficient contrast, and some noise; (4) good image quality, good contrast, and little noise; and (5) excellent image quality, good contrast, and no significant noise. The initial window width and position were set at 350 and 50 HU, respectively, and both parameters were modifiable.

### Radiation dose

To evaluate radiation dose, we recorded the volume CT dose index (CTDIvol) and dose length product (DLP) on the scanner and calculated the effective radiation dose. The effective radiation dose corresponded to the value of the DLP multiplied by the abdominal conversion coefficient, which was 0.015 mSv/mGy.cm.

### Statistical analysis

All analyses were performed using SPSS version 25.0 (IBM Corp., Armonk, NY, USA). The Kolmogorov–Smirnov test was used to determine whether the data were normally distributed. LD-HIR images were used as references to assess the image quality, renal-calculus measurements, radiation exposure, and lesion detection on ULDCT. We used the Kruskal–Wallis nonparametric test to analyze multigroup differences and the Mann–Whitney *U* nonparametric test for pairwise comparisons. *p* < 0.05 was considered statistically significant. We used Cohen’s weighted *κ *to calculate interobserver agreement, scored as almost perfect (0.81–1.00), substantial (0.61–0.80), moderate (0.41–0.60), fair (0.21–0.40), or poor (0.00–0.20).

## Results

In this prospective study, 60 patients (35 men and 25 women) with a mean ± SD age of 50.7 ± 13.5 (range, 27–81) years were enrolled. One patient underwent splenectomy, and another underwent left nephrectomy.

### Detection of calculi and abdominal lesions

**Fig. 2:**
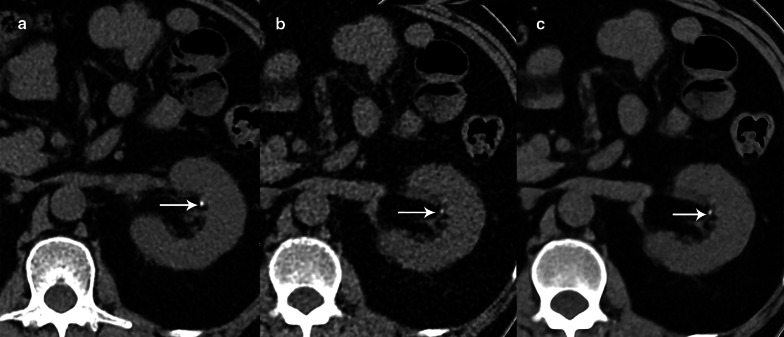
CT images from an 81-year-old female patient with a clinically suspected renal calculus. A left renal calyceal calculus 1.9 mm in diameter was detected on all the three images. However, LD-HIR (**a**) was clearest, followed by ULD-DLR (**c**), while ULD-HIR (**b**) was the least clear. LD-HIR means low-dose computed tomography with hybrid iterative reconstruction. ULD-HIR means ultra-low-dose computed tomography with hybrid iterative reconstruction. ULD-DLR means ultra-low-dose computed tomography with deep learning reconstruction

**Fig. 3:**
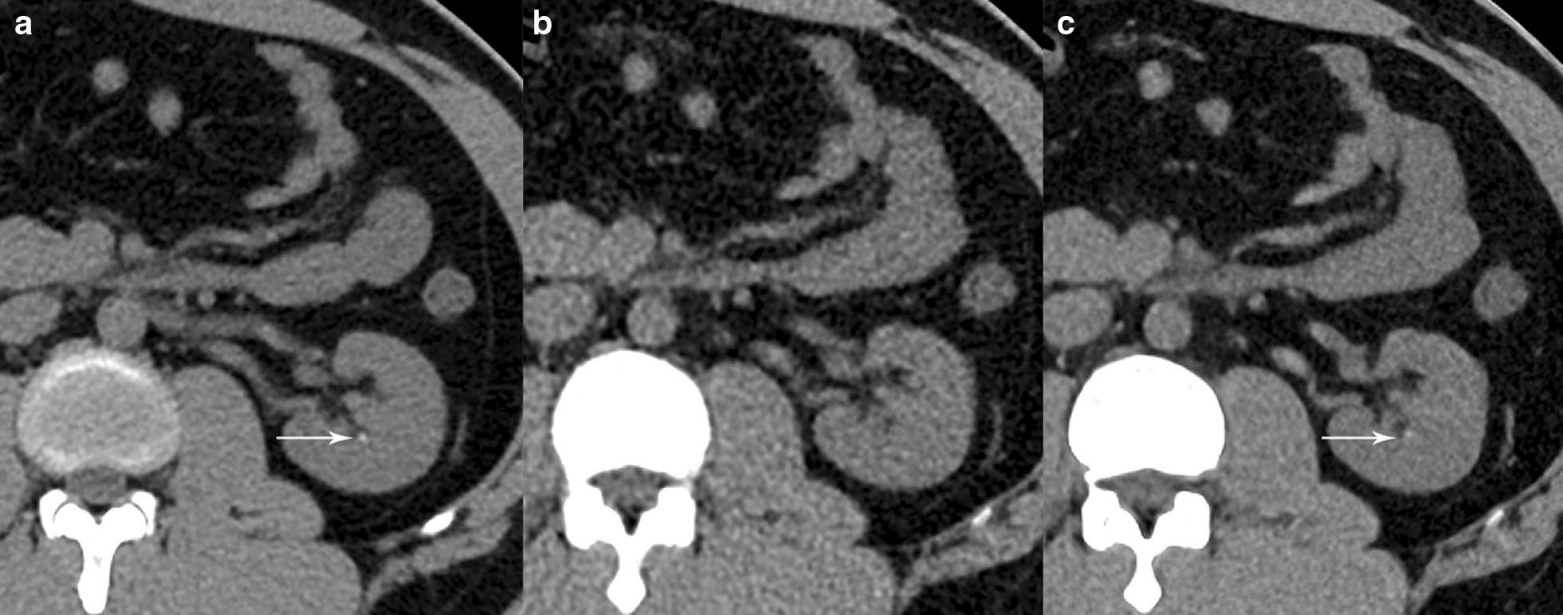
CT images from a 65-year-old male patient with a clinically suspected renal calculus. A left renal calyceal calculus 1.5 mm in diameter was visible on the LD-HIR (**a**) and ULD-DLR (**c**) images but not on the ULD-HIR (**b**) image. LD-HIR means low-dose computed tomography with hybrid iterative reconstruction. ULD-HIR means ultra-low-dose computed tomography with hybrid iterative reconstruction. ULD-DLR means ultra-low-dose computed tomography with deep learning reconstruction

**Fig. 4:**
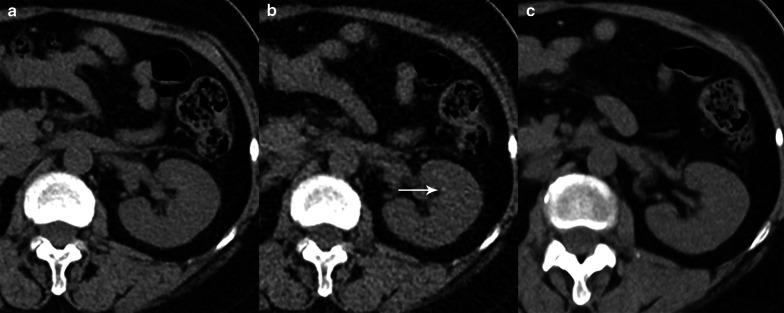
CT images from a 57-year-old male patient with a clinically suspected renal calculus. A left renal calyceal calculus 1.6 mm in the diameter could be seen on the ULD-HIR (**b**) image but not on the LD-HIR (**a**) or ULD-DLR (**c**) images. With LD-HIR imaging used as the gold standard, the stone was a false positive. LD-HIR means low-dose computed tomography with hybrid iterative reconstruction. ULD-HIR means ultra-low-dose computed tomography with hybrid iterative reconstruction. ULD-DLR means ultra-low-dose computed tomography with deep learning reconstruction

On the LD-HIR images, 130 renal calculi (left kidney, 72; right kidney, 58) were detected. The mean ± SD diameter of the renal calculi was 5.16 ± 4.20 mm. Six renal calculi with a mean diameter of 1.93 mm were missed on the ULD-DLR images. Nine renal calculi with a mean diameter of 1.96 mm were missed on the ULD-HIR images, including the 6 stones missed on ULD-DLR images. Taking the LD-HIR images as a reference, the renal-calculus detection rates of ULD-HIR and ULD-DLR were respectively 93.1% and 95.4% for all renal calculi, and the rates were 100% for stones measuring > 3 mm. The mean diameters of the renal calculi measured using ULD-HIR and ULD-DLR were 5.39 ± 4.37 mm and 5.33 ± 4.33 mm, respectively. There was no statistical difference in calculus size among the 3 groups (*p* > 0.05; Figs. 2 and 3). The diameters of the smallest calculi observed on LD-HIR, ULD-HIR and ULD-DLR were 1.5 mm, 1.7 mm and 1.5 mm, respectively. No false positives were detected on ULD-DLR images, but one stone 1.6 mm in diameter was found to be a false positive on ULD-HIR images (Fig. 4).

We detected 129 lesions on the LD-HIR images, including 34 hepatic cysts, 74 renal cysts, 11 hepatic calcifications, 2 hypodense hepatic lesions, 2 gallstones, 2 splenic cysts, 2 adrenal adenomas, 1 hypodense spleen lesion, and 1 pancreatic cyst. The lesion detection rate on ULD-DLR images was 92.2% (119/129). Except for 4 hepatic cysts measuring < 5 mm and 6 renal cysts measuring < 5 mm, all lesions detected via LD-HIR were observable on ULD-DLR images. The lesion detection rate for ULD-HIR images was 82.9% (107/129). Eight hepatic cysts with an average size of 7.63 mm, 11 renal cysts with an average size of 8.51 mm, 1 hypodense hepatic lesion 10.6 mm in size, 1 hypodense spleen lesion 11.9 mm in size, and 1 pancreatic cyst 12.3 mm in size could not be observed on ULD-HIR images.

### Objective image quality

The objective parameters of image quality, including CT values, image noise, CNR, and SNR, in various abdominal organs and tissues on LD-HIR, ULD-HIR, and ULD-DLR images are shown in Tables 1, 2 and 3.

CT values of various tissues and organs did not differ to a statistically significant degree between ULD-DLR and LD-HIR images (*p* > 0.05). CT values of all tissues and organs obtained via ULD-HIR, except for fat, were higher than those obtained via LD-HIR images (*p* < 0.05). CT values of the liver and aorta on ULD-HIR images were similar to those on ULD-DLR images (*p* > 0.05), but CT values of other tissues and organs were higher than on ULD-DLR images (*p* < 0.05; Table 1).

**Table 1:** CT values for various abdominal organs and tissues

CT (HU)	LD-HIR (group 1)	ULD-HIR (group 2)	ULD-DLR (group 3)	P 1 vs. 2 vs. 3	P 1 vs. 2	P 1 vs. 3	P 2 vs. 3
Liver	56.30 ± 8.87	59.44 ± 8.32	57.70 ± 8.36	0.104	0.034	0.389	0.216
Spleen	50.16 ± 3.39	52.83 ± 3.32	51.11 ± 3.29	< 0.001	< 0.001	0.144	0.005
Kidney	32.89 ± 3.46	35.38 ± 3.87	33.73 ± 3.69	< 0.001	< 0.001	0.097	0.003
Aorta	43.47 ± 5.90	46.01 ± 5.23	44.81 ± 4.90	0.025	0.008	0.173	0.137
Muscle	50.76 ± 5.35	54.94 ± 7.00	51.57 ± 6.09	< .001	< 0.001	0.221	< 0.001
Fat	− 110.11 ± 9.73	− 111.02 ± 6.69	− 108.48 ± 6.51	0.064	0.848	0.074	0.025

There were no statistically significant differences in CNRs for the liver, kidneys, spleen, and aorta between ULD-DLR and LD-HIR images (*p* > 0.05). The CNRs of the aorta and spleen on ULD-HIR images were not statistically significantly different from those on LD-HIR images (*p* > 0.05), but the CNRs of the liver and kidneys on ULD-HIR were lower than those on LD-HIR images (*p* < 0.05). The CNRs of the four organs obtained via ULD-HIR, except for the aorta, were lower than those obtained via ULD-DLR images (*p* < 0.05) (Table 2).

**Table 2:** Contrast-to-noise ratios (CNRs) for the liver, spleen, kidneys, and aorta

CNR	LD-HIR (group 1)	ULD-HIR (group 2)	ULD-DLR (group 3)	P 1 vs. 2 vs. 3	P 1 vs. 2	P 1 vs. 3	P 2 vs. 3
Liver	1.18 ± 0.84	0.77 ± 0.65	1.24 ± 0.99	< 0.001	0.005	0.290	0.003
Spleen	0.57 ± 0.47	0.55 ± 0.49	0.72 ± 0.58	0.148	0.696	0.142	0.034
Kidney	2.30 ± 0.76	1.69 ± 0.62	2.40 ± 0.83	< 0.001	< 0.001	0.564	< 0.001
Aorta	1.04 ± 0.72	0.94 ± 0.62	1.13 ± 0.74	0.393	0.596	0.515	0.146

Anterior abdominal-wall subcutaneous fat SD was used to estimate total noise on LD-HIR, ULD-HIR, and ULD-DLR images, and the values were 7.97 ± 1.25 HU, 12.14 ± 2.13 HU, and 7.58 ± 1.67 HU, respectively. No significant difference in total image noise existed between LD-HIR and ULD-DLR images (*p* > 0.05), and both had lower total image noise than ULD-HIR images (*p* < 0.05). Of the three groups, the ULD-DLR images had the highest SNR and the lowest noise; while the ULD-HIR images had the lowest SNR and the highest noise (*p* < 0.05; Table 3).

**Table 3:** Noise and signal-to-noise ratios (SNRs) for various abdominal organs and tissues

Organ	LD-HIR (group 1)	ULD-HIR (group 2)	ULD-DLR (group 3)	P 1 vs. 2 vs. 3	P 1 vs. 2	P 1 vs. 3	P 2 vs. 3
*Noise (HU)*							
Liver	9.10 ± 0.91	14.60 ± 1.72	7.57 ± 0.49	< 0.001	< 0.001	< 0.001	< 0.001
Spleen	9.25 ± 1.11	14.14 ± 1.39	7.40 ± 0.57	< 0.001	< 0.001	< 0.001	< 0.001
Kidney	9.18 ± 1.17	14.06 ± 2.08	7.31 ± 0.81	< 0.001	< 0.001	< 0.001	< 0.001
Aorta	9.73 ± 1.52	14.35 ± 2.11	7.84 ± 1.08	< 0.001	< 0.001	< 0.001	< 0.001
Muscle	10.04 ± 1.52	14.85 ± 2.57	7.52 ± 1.04	< 0.001	< 0.001	< 0.001	< 0.001
Fat	7.98 ± 1.25	12.14 ± 2.13	7.58 ± 1.67	< 0.001	< 0.001	0.053	< 0.001
*SNR*							
Liver	6.27 ± 1.29	4.11 ± 0.73	7.69 ± 1.29	< 0.001	< 0.001	< 0.001	< 0.001
Spleen	5.50 ± 0.82	3.79 ± 0.42	6.94 ± 0.68	< 0.001	< 0.001	< 0.001	< 0.001
Kidney	3.65 ± 0.72	2.56 ± 0.40	4.60 ± 0.53	< 0.001	< 0.001	< 0.001	< 0.001
Aorta	4.56 ± 0.90	3.28 ± 0.63	5.83 ± 1.08	< 0.001	< 0.001	< 0.001	< 0.001
Muscle	5.17 ± 0.99	3.81 ± 0.81	7.04 ± 1.39	< 0.001	< 0.001	< 0.001	< 0.001
Fat	14.18 ± 2.72	9.74 ± 3.51	14.69 ± 2.44	< 0.001	< 0.001	0.268	< 0.001

### Subjective image quality

Table 4 shows subjective image quality scores for the three groups of images. Both reviewers deemed the quality of ULD-DLR images to be similar to that the LD-HIR images (*p* > 0.05 for both reviewers), while ULD-HIR image scores were significantly lower than those of LD-HIR and ULD-DLR images (*p* < 0.05 for both reviewers). Image quality interobserver agreement was substantial for LD-HIR images (*κ* = 0.63), moderate for ULD-HIR images (*κ* = 0.48) and perfect for ULD-DLR images (*κ* = 0.83).

**Table 4:** Subjective-quality scores of LD-HIR, ULD-HIR, and ULD-DLR images

Group	Image quality												Kappa value
	Reader 1						Reader 2						
Score	1	2	3	4	5	mean	1	2	3	4	5	mean	
LD-HIR	0	0	0	23	37	4.57	0	0	0	21	39	4.62	0.63
ULD-HIR	0	5	43	12	0	3.12	0	1	49	9	1	3.17	0.48
ULD-DLR	0	0	0	31	29	4.55	0	0	1	27	33	4.52	0.83

### Radiation dose

CTDIvol was 3.42 ± 0.52 mGy for LDCT and 0.80 ± 0.19 mGy for ULDCT. DLP was 183.15 ± 33.55 mGy.cm for LDCT and 42.73 ± 11.23 mGy.cm for ULDCT. Effective radiation doses were 2.75 ± 0.50 mSv and 0.64 ± 0.17 mSv for LDCT and ULDCT, respectively. Notably, the effective radiation dose for ULDCT was 77% lower than that for LDCT.

## Discussion

This study showed that when the scan radiation dose was decreased to sub-millisievert ultra-low doses, the detection rate obtained by ULD-DLR for kidney stones > 3 mm in diameter was 100% in reference to LD-HIR images. On ULD-DLR images, 4 hepatic cysts and 6 renal cysts with diameters < 5 mm were missed. There was no significant reduction in diagnostic efficiency in either kidney stones or abdominal lesions. At the same time, ULD-DLR images had higher image quality than LD-HIR and ULD-HIR images. The radiation dose used for ULDCT was < 1 mSv, a significant (77%) decrease over that for LDCT. This was the equivalent of the dose used for a single abdominal x-ray [28].

Renal calculi commonly occur and tend to recur, requiring radiological examination for diagnostic confirmation and monitoring. Currently, LDCT scans combined with iterative reconstruction algorithms are routinely used for renal-calculus detection and confirmation, with a 100% detection rate for renal calculi > 3 mm in diameter [13, 29]. To further reduce radiation doses, emerging studies are assessing kidney stones using ULDCT. A systematic review showed that ULDCT (< 1.9 mSv) and LDCT (< 3.5 mSv) show comparable sensitivity and specificity in urolithiasis detection [30]. Two studies, one by Roberts et al. involving 21 patients and another by McLaughlin et al. involving 33 patients, reported no significant differences in renal-calculus detection rate or size limit between ULDCT and LDCT when the radiation dose was decreased to sub-millisievert levels [14, 28]. Our study obtained similar results, supporting the use of ULDCT for renal-calculus detection and monitoring at a much lower radiation dose. Our study included more renal calculi than previous studies with ULD scans. In our study, 130 calculi were evaluated, and only 6 calculi with an average diameter of 1.93 mm were missed on ULD-DLR images. Because these missed calculi were too small, their CT values measurement is not accurate, so the CT values of missed calculi were not analyzed. Clinically, calculi of this size rarely require therapeutic intervention because most stones < 6 mm can be spontaneously discharged [4, 31]. Therefore, missed diagnoses of small renal calculi on ULD-DLR images do not cause adverse clinical outcomes. In addition, these previous studies used iterative reconstruction, which posed limitations to soft-tissue analysis when the radiation dose was decreased to sub-millisievert levels [14, 28]. Therefore, existing studies on ULDCT for kidney stones offer only limited assessment of image quality and detection of other abdominal lesions. Roberts et al. evaluated the detection of renal masses only [28], and McLaughlin et al. evaluated organ noise only [14], Another advantage of performing CT scans on renal-calculus patients is that detailed information on anatomical structures can be obtained for the detection of other abdominal diseases. However, the higher noise level can affect diagnostic performance. Therefore, in addition to evaluating renal-calculus detection, in this study, we also examined the possibility of maintaining low-noise levels when DLR is used for image reconstruction in ULDCT, with the aim of reducing the radiation dose.

In addition, we evaluated objective and subjective image quality, as well as lesion detection rates for the liver, kidneys, spleen, and aorta on ULD-DLR images. The results showed that detection rates for abdominal lesions obtained via ULD-DLR were comparable to those obtained via LDCT because missing a diagnosis of cysts that < 5 mm in diameter has no serious effect on clinical diagnosis. Phantoms and clinical studies have shown that DLR, the new reconstruction method, could further improve image quality and decrease CT image artifacts compared with conventional iterative reconstruction when used with LDCT of the coronary artery, chest, and abdomen [20, 21, 23, 24, 27]. These studies also show that DLR has good prospects for decreasing CT radiation dose and does not significantly increase the reconstruction duration. Nakamura et al. found that DLR can decrease radiation dose in high-resolution abdominal CT to 70% of the standard dose without reducing image quality [32]. Singh et al. further demonstrated that DLR can fundamentally maintain objective image quality and detect low-contrast lesions in the liver when sub-millisievert scans are performed on the chest and abdomen; the radiation dose can be decreased to 71% of that used for low-dose scans [33]. However, current studies on DLR of the abdomen have mainly focused on low-contrast lesions such as in the liver, with almost no evaluation of high-contrast renal calculi combined with ULDCT [34]. In this study, we also assessed kidney stones and abdominal disease, and the results indicated that DLR had high diagnostic performance for both high- and low-contrast lesions on ULDCT. Therefore, this study enhances current knowledge on the usability of sub-millisievert scans for the detection of renal calculi and the usability of DLR in ULDCT for abdominal diseases.

There were some limitations to our study. First, patients received additional radiation during the second abdominal ULDCT scan. Nevertheless, the mean effective dose of the ultra–low-dose protocol (0.64 mSv) was relatively low, and the cumulative dose from both protocols was 1.95–4.90 mSv, falling within the range of the radiation dose used for renal colic CT imaging [35]. Second, only HIR and DLR reconstructions were employed; we did not consider model-based iterative reconstruction (MBIR) because it is not routinely used at our hospital, has a long reconstruction duration, and, as shown by previous studies, results in lower image quality than DLR reconstruction when radiation dose is low [23, 27]. Third, this study mainly focused on suspected renal calculus patients and, therefore, the scan area was limited to the abdomen. In some patients with suspected renal calculi, stones might travel to the ureter and bladder, which will be considered in future studies by our group.

In summary, a combination of ULDCT and DLR reconstruction could ensure renal calculus detection, decrease image noise, and improve image quality. This methodology can maintain image quality while greatly decreasing radiation dose for the clinical diagnosis and monitoring of renal calculi.

## Data Availability

All data is presented in this work.

## Abbreviations

CNR: Contrast-to-noise ratio
CT: Computed tomography
CTDIvol: CT dose index
DLP: Dose length product
DLR: Deep learning reconstruction
HIR: Hybrid iterative reconstruction
LDCT: Low-dose computed tomography
ROI: Region of interest
SNR: Signal-to-noise ratio
ULDCT: Ultra-low-dose computed tomography
